# Complete Genome Sequence of the *Streptomyces*-Specific Bacteriophage BRock

**DOI:** 10.1128/MRA.00624-20

**Published:** 2020-08-27

**Authors:** Stephen F. Baron, Ashley N. Crossman, Shikha Malik, Parveen Sidhu, Kiran Nehra, Pragati Jamdagni, Ivan Erill, Louise M. Temple

**Affiliations:** a Department of Biology, Bridgewater College, Bridgewater, Virginia, USA; b Virginia-Maryland College of Veterinary Medicine, Virginia Tech, Blacksburg, Virginia, USA; c Deenbandhu Chhotu Ram University of Science and Technology, Murthal, Haryana, India; d Department of Biological Sciences, University of Maryland Baltimore County, Baltimore, Maryland, USA; e School of Integrated Sciences, James Madison University, Harrisonburg, Virginia, USA; Queens College

## Abstract

The complete genome sequence of the unique virulent bacteriophage BRock, isolated from compost on *Streptomyces* sp. strain SFB5A, was determined. BRock is a myovirus with a 112,523-bp genome containing a GC content of 52.3%. There were 188 protein-coding genes predicted, including structural and enzymatic proteins, but none predicted for lysogeny. Twenty-nine tRNAs were predicted.

## ANNOUNCEMENT

The genus *Streptomyces* consists of Gram-positive, filamentous environmental bacteria with a GC content of ∼70%. Because these bacteria produce abundant antibiotics (1), there is considerable interest in isolating *Streptomyces*-specific bacteriophages. To date, 882 *Streptomyces*-specific bacteriophages in 17 distinct clusters have been isolated and 242 of their genomes sequenced (https://phagesdb.org/clusters/).

Bacteriophage BRock (https://phagesdb.org/phages/BRock/) was isolated in January 2016 from compost (GPS coordinates 38.37N, 78.96W) after amplification with *Streptomyces* sp. strain SFB5A (NCBI taxonomy identifier 2663842) in tryptic soy broth plus 4 mM CaCl_2_ (TSB-Ca) at 30°C with agitation. This procedure was followed by double-layer agar plating (2) using TSB-Ca top agar (0.6%) overlaid onto Trypticase soy agar (30°C) and three subsequent rounds of plaque purification.

Phage genomic DNA was prepared with the Wizard DNA clean-up system (Promega, Madison, WI, USA). Sequencing libraries were prepared with an Illumina TruSeq Nano DNA library prep kit, yielding 350-bp inserts (quality control with a TapeStation D1000 system) and run on the Illumina MiSeq platform. Raw data were assembled with Newbler 2.1 (3) into one contig with 200-fold coverage from a 100,000-read random subset generated with seqtk version 1.1-r91 (https://github.com/lh3/seqtk/releases/tag/v1.1). Protein gene prediction and annotation were performed using DNA Master version 5.22.5 (http://cobamide2.bio.pitt.edu/) and tRNA prediction with ARAGORN (4).

The BRock genome had 112,523 bp, a GC content of 52.3%, and a 221-bp direct terminal repeat indicated by double coverage with well-defined margins in the assembled contig. There were 188 protein-coding sequences, including 40 with assigned functions. The absence of recombination genes indicated that BRock may be virulent. There were 29 predicted tRNA genes, representing all 20 naturally occurring amino acids.

**FIG 1:**
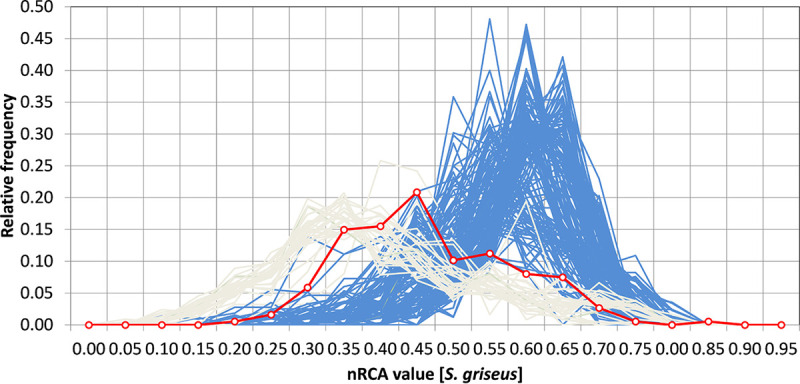
Distributions of nRCA scores (7) for all protein-coding genes in the genomes of 192 *Streptomyces*-infecting phages (i.e., codon usage bias score, model trained on *S. griseus* ribosomal genes). The subset of 28 phages including phage BRock (see the text) is shown in light gray, with phage BRock itself highlighted in red with open circles. The other 164 phages are shown in blue.

GLIMMER, a self-training model (5), predicted 188 protein-coding genes while GeneMark.hmm (6), trained using Streptomyces griseus as a model, predicted only 85. The 103 genes not called by GeneMark.hmm had a lower GC content than the rest of the genome and did not occur in a single region or a few large regions that might suggest some horizontal transfer events or a hybrid phage. To explore these observations, codon usage bias was analyzed on 192 *Streptomyces* phage genomes, using the normalized relative codon adaptation (nRCA) index (7) with *S. griseus* ribosomal proteins as a reference set and otherwise default parameters (Fig. 1). The nRCA index was computed using Python scripts. These scripts, as well as a file containing the NCBI nucleotide accession numbers for all genomes analyzed, are available online at https://github.com/ErillLab/nRCA. BRock belonged to a subgroup of 28 phages with a GC content of <53%, which is 1.5 standard deviations below the mean (62.90%) for all phages analyzed in this work, considerably lower than that of *Actinomycetales*-infecting phages and substantially lower than that of *Streptomyces* hosts (∼70%). These 28 phages are larger (∼130,800 bp) than the other 164 phages (∼50,632 bp) and have lower codon usage bias values (nRCA, ∼0.43 versus 0.6). They also possess multiple predicted tRNA genes (36 to 46), whereas the other 164 phages predominantly lack predicted tRNA genes. Collectively, these findings may explain the differences in results obtained with the two gene-calling programs. Furthermore, blastn analysis (8) of the BRock genome showed no significant homology to any other *Actinomycetales* phage genomes, including *Streptomyces* phage clusters BE and BK, which are the most similar to BRock in genome size and GC content.

### Data availability.

The complete genome sequence of the bacteriophage BRock has been deposited in GenBank under the accession number KX925554.1. The version described in this paper is the first version. The short-read sequences have been deposited under BioProject number PRJNA630487, BioSample number SAMN15032879, and Sequence Read Archive number SRP265028.
